# Primary Renal Small Cell Neuroendocrine Carcinoma With Inferior Vena Cava Tumor Thrombus: A Report of a Rare Case

**DOI:** 10.7759/cureus.85693

**Published:** 2025-06-10

**Authors:** Duy T Nguyen, Hoang X Nguyen, Phuc T Hoang

**Affiliations:** 1 Department of Urology, Binh Dan Hospital, Ho Chi Minh, VNM

**Keywords:** intratumoral pseudoaneurysm, neuroendocrine tumours (nets), primary renal carcinoid, renal neuroendocrine tumor, tumor thrombus

## Abstract

Primary renal neuroendocrine tumors (NETs) are exceedingly rare. Among these, small cell neuroendocrine carcinoma (SCNC) represents an aggressive and poorly differentiated subtype, with very few documented cases. We present a case of a 24-year-old male patient who presented with gross hematuria and flank pain. Imaging revealed a large right renal mass with a tumor thrombus extending into the inferior vena cava (IVC). The patient underwent radical nephrectomy, adrenalectomy, and IVC thrombectomy. Histopathological examination confirmed a high-grade small cell neuroendocrine carcinoma with extensive necrosis and vascular invasion. Immunohistochemistry was positive for CD56, synaptophysin, and a high Ki-67 index (~70%). Despite radical surgery and systemic chemotherapy with carboplatin and etoposide, the patient developed liver metastases within three months and succumbed to disease progression nine months postoperatively. This case underscores the rarity and aggressive nature of primary renal SCNC, the clinical significance of IVC tumor thrombus as a poor prognostic factor, and the rapid disease progression that can occur even in young patients. Multimodal therapy remains the mainstay of treatment, but the overall prognosis is poor. Further investigation into molecular characteristics and targeted therapies is urgently needed.

## Introduction

Neuroendocrine tumors (NETs) are rare neoplasms, most commonly occurring in the lungs or gastrointestinal tract (accounting for approximately 20-50% of NET cases), whereas primary renal NETs are exceedingly rare, comprising less than 1% of renal tumors [1-3]. Primary renal NETs include well-differentiated carcinoids and poorly differentiated variants such as small cell and large cell neuroendocrine carcinomas [2,4]. Most patients are asymptomatic or present with specific symptoms such as hematuria, flank pain, or an abdominal mass [5,6].

According to the World Health Organization (WHO) classification, NETs are graded based on Ki-67 index and differentiation, ranging from well-differentiated carcinoid tumors to poorly differentiated small cell neuroendocrine carcinoma (SCNC), characterized pathologically by small round cells arranged in nests or rosettes with extensive necrosis [7]. Unlike conventional renal cell carcinoma, primary renal NETs often demonstrate early metastasis to lymph nodes, liver, or bone, even in well-differentiated cases [3,8-10]. Their biological behavior is unpredictable, and prognosis remains guarded, especially in the presence of high-grade features, advanced stage, or metastatic disease [1,3,4]. SCNC is often chemoresistant, with poor response and a median progression-free survival of three to six months, necessitating further case reports to optimize treatment strategies [1,8].

The presence of tumor thrombus extending into the inferior vena cava (IVC) is extremely rare, with only a few reported cases in the literature [10-12]. Such vascular involvement significantly complicates surgical management and may portend a poorer outcome. This report highlights a rare case of primary renal NET with IVC tumor thrombus and distant metastasis.

## Case presentation

A 24-year-old male patient presented with gross hematuria and dull right flank pain persisting for two months. The patient had no history of fever, dysuria, or systemic symptoms. The patient had no prior history of medical comorbidities or previous surgical interventions. Family history was negative for malignancies. Physical examination revealed a palpable, firm, non-tender mass measuring approximately 15×10 cm, located in the right hypochondriac region. He remained hemodynamically stable, with a Karnofsky performance score of 90.

**Figure 1:**
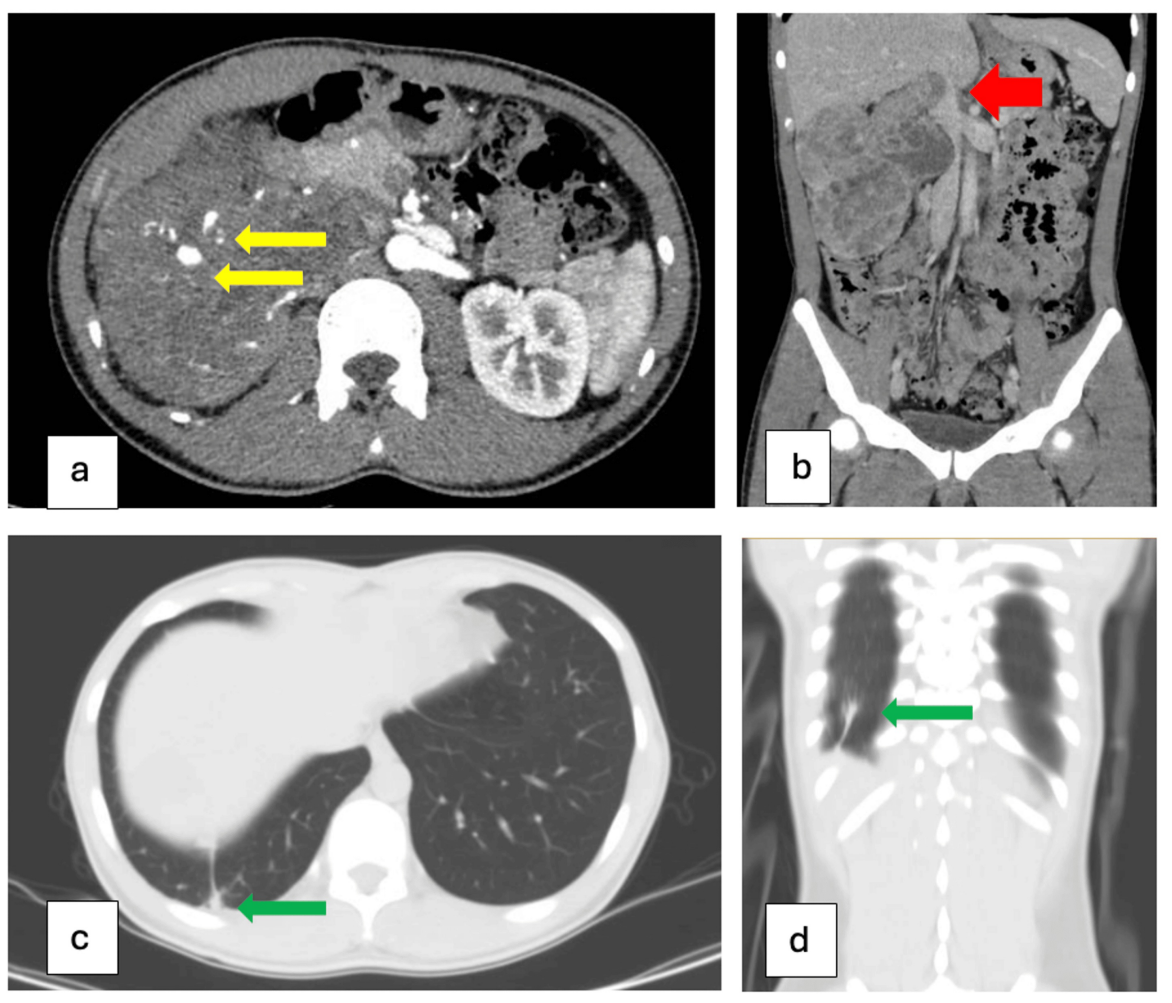
Preoperative MSCT imaging of right renal mass with IVC tumor thrombus and a pulmonary node. (a) Axial arterial phase CT demonstrates a large, heterogeneously enhancing right renal mass measuring 94×126×151 mm, with evidence of perinephric fat invasion. Yellow arrows highlight intratumoral pseudoaneurysms. (b) Coronal portal venous phase CT image demonstrates the tumor exerts a mass effect on the adjacent IVC, resulting in compression and displacement. A contiguous, enhancing soft tissue component within the IVC is visualized, consistent with a tumor thrombus extending from the renal mass (red arrow). (c) Axial chest CT reveals a consolidation lesion in segment 10 of the right lung (green arrow), measuring 25×15×16 mm, suspicious for a benign node. (d) Coronal CT of the chest confirms the location of the pulmonary lesion in the posterior basal segment of the right lower lobe (green arrow). MSCT: multislice computed tomography; IVC: inferior vena cava.

Preoperative contrast-enhanced multislice computed tomography (MSCT) showed a large, heterogeneously enhancing right renal mass measuring 94×126×151 mm with perinephric fat invasion, contact with the right liver margin, and abdominal wall. There are several pseudoaneurysms within the tumor measured 13x10 mm and an IVC tumor thrombus measuring up to 24 mm in diameter. The thrombus extends into the IVC, reaching 15 mm above the renal vein orifice, which corresponds to Level I according to the Mayo Clinic classification for IVC tumor thrombus (Figure 1).

The mass appeared to involve the adrenal gland, and several small, scattered lymph nodes were observed in the surrounding regions. Chest CT revealed a consolidation lesion in segment 10 of the right lung (25×15×16 mm) suspected to present a benign node with no association with pleural effusion or mediastinal lymphadenopathy noted. Additionally, cranial CT was unremarkable and blood workup revealed normal renal and liver function, with moderate anemia according to the WHO classification (Table 1).

**Table 1:** Perioperative blood test and renal function. WBCs: white blood cells; RBCs: red blood cells; PLT: platelet count; eGFR: estimated glomerular filtration rate; MDRD: modification of diet in renal disease.

Parameter	Two weeks before surgery	Five days before surgery	Postoperative day 1	Postoperative day 2	Reference range
WBCs	10.1	9.7	10.1	9.8	4.6-10 (K/µL)
RBCs	5.1	4.1	3.65	3.45	4.09-5.74 (M/µL) (male)
Hemoglobin	13.4	10.8	9.4	8.9	13.1-17.2 (g/dL) (male)
Hematocrit	41.9	33.7	30.4	27.5	41-53 (%) (male)
PLT	392	349	338	285	142-424 (K/µL)
Urea	4.2	-	3.5	2.6	2.1-8.2 (mmol/L)
Creatinine	87	-	65	82	44-115 (µmol/L)
eGFR (MDRD)	93.5	-	130.9	100.1	>60 (ml/min/1.73m^2^)

Approximately one week prior to surgery, the patient developed worsening abdominal pain, accompanied by gross hematuria and complete loss of appetite. These new symptoms prompted urgent clinical reassessment. After a multidisciplinary tumor board discussion, urgent surgical intervention was recommended, and the patient was scheduled for operation.

**Figure 2:**
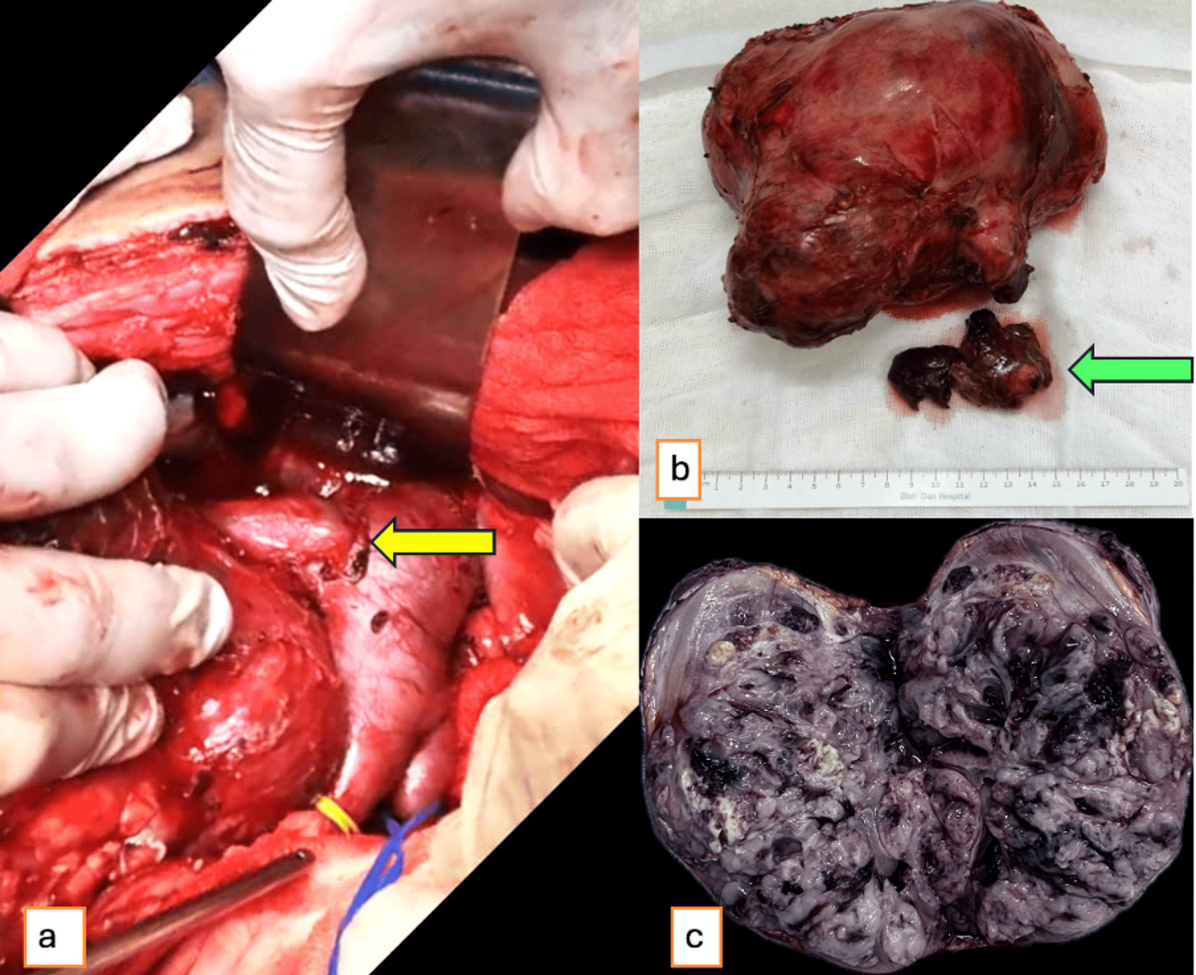
Right radical nephrectomy with IVC thrombectomy. (a) Intraoperative view after exposure of a large right renal tumor and the adjacent organs have been mobilized to facilitate visualization. The right renal vein is indicated by a yellow arrow, IVC is encircled with a yellow vessel loop, while the contralateral (left) renal vein is marked with a blue loop. (b) A gross specimen of the resected right kidney with the tumor measured approximately 18×15 cm, thrombus was retrieved from the renal vein/IVC (green arrow). (c) Cut section of the tumor showing heterogeneous tumor with intratumoral hematoma. IVC: inferior vena cava.

Surgery was performed via a midline laparotomy under general anesthesia. A right radical nephrectomy, right adrenalectomy, and IVC thrombectomy were performed with an operative time of 240 minutes. Intraoperatively, the tumor measured approximately 18×15 cm with a nodular surface and dense adhesions to the perirenal tissue (Figure 2). The IVC thrombus extended 3 cm above and 2 cm below the entry of the right renal vein, confirmed by intraoperative Doppler ultrasound. The IVC was clamped above and below the thrombus, opened longitudinally, and the tumor thrombus (4×2 cm) was successfully removed without vascular wall invasion. The IVC was reconstructed using 5-0 Prolene (CPT Sutures Co., Ltd., Ho Chi Minh City, Vietnam) sutures. Total blood loss was estimated at 1000 ml.

**Figure 3:**
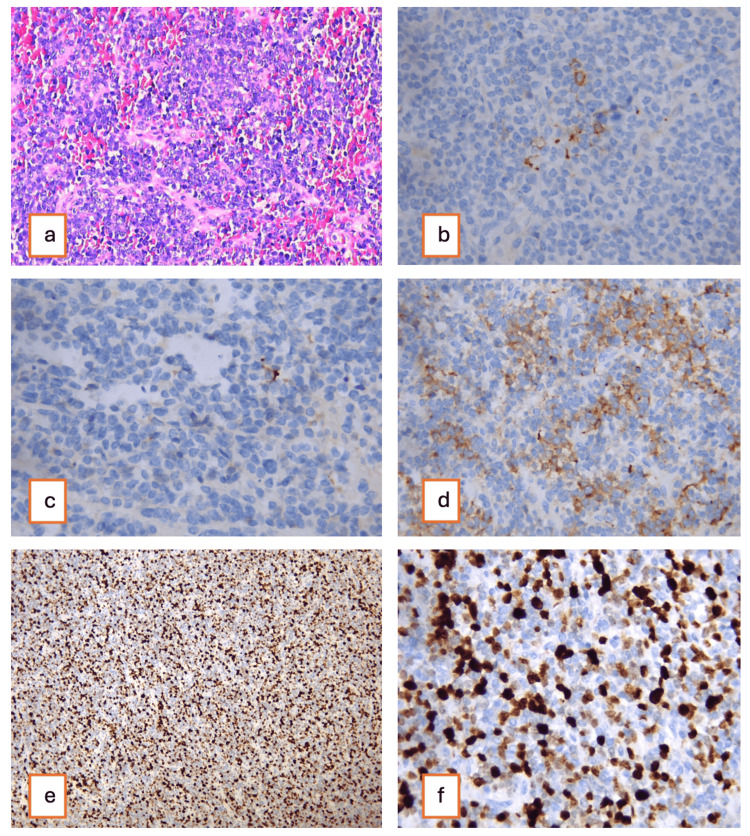
Histopathological and immunohistochemical features of high-grade small cell neuroendocrine carcinoma. Hematoxylin and eosin (H&E) stain shows small round tumor cells in nests and rosettes with scant cytoplasm and fine chromatin (a). Immunohistochemistry (40x magnification) shows CD56 positivity (b), focal weak CK expression (c), and synaptophysin positivity (d). A high Ki-67 proliferation index of approximately 70%, is shown at 10x magnification (e) and 40x magnification (f).

Pathological evaluation demonstrated that the tumor was composed of small round cells arranged in nests and rosettes, with scant cytoplasm and finely granular chromatin. Extensive necrosis and vascular invasion, including renal vein and IVC thrombus, were observed. Immunohistochemistry showed CD56 and synaptophysin positivity, weak CK staining, and a high Ki-67 index (around 70%) (Figure 3). The final diagnosis was high-grade small cell neuroendocrine carcinoma of the kidney with vascular invasion.

Postoperatively, the patient gradually recovered with significant pain relief and resumption of normal daily activities. No gross hematuria or abnormal bleeding was observed. Despite undergoing a right nephrectomy, renal function remained preserved, with stable creatinine and eGFR values, reflecting adequate compensation by the remaining left kidney and no signs of acute kidney injury (Table 1). He was discharged in stable condition on postoperative day 8. Systemic chemotherapy was initiated in the second month after surgery, following a standard combination regimen of carboplatin and etoposide (six cycles, every 21 days), as indicated for extrapulmonary small cell neuroendocrine carcinoma.

**Figure 4:**
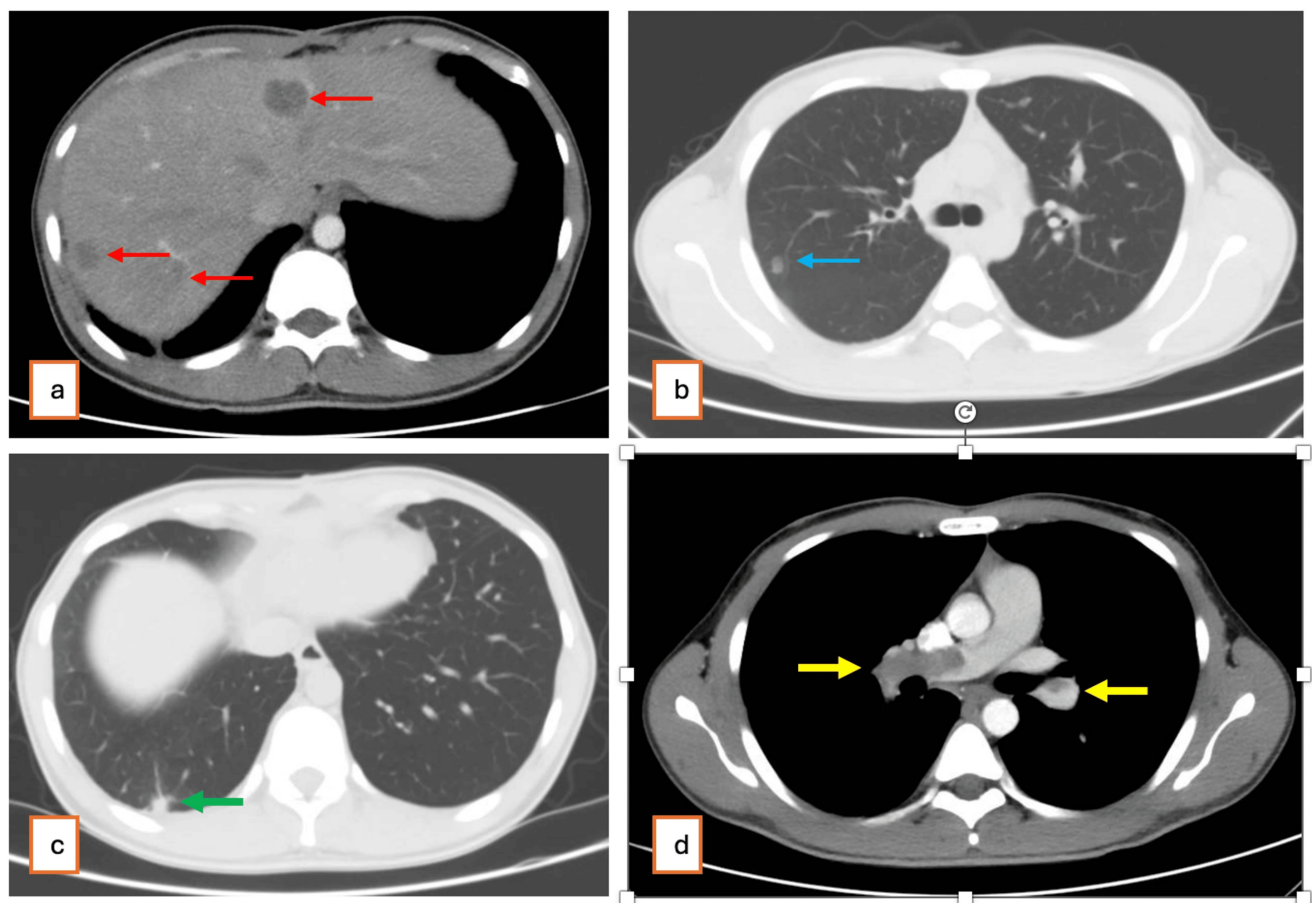
Contrast-enhanced MSCT at three months after surgery. (a) Axial portal venous phase CT shows multiple hypodense lesions in both hepatic lobes (red arrows), consistent with secondary liver metastases. (b) Axial chest CT demonstrates a new pulmonary nodule in the right lung (blue arrow), suspicious for metastatic disease. (c) Axial chest CT shows a pulmonary lesion (green arrow) that was present on the preoperative scan, with no significant change in size or morphology, suggesting stability. (d) Arterial phase imaging demonstrates partial occlusive thrombosis of both the right and left main pulmonary arteries (yellow arrows). MSCT: multislice computed tomography.

However, follow-up contrast-enhanced MSCT performed three months after surgery revealed disease progression with multiple hypodense lesions in both hepatic lobes suggestive of secondary liver metastases, scattered retroperitoneal and right renal fossa deposits, bilateral pulmonary nodules, and partial occlusion of both the right and left main pulmonary artery (Figure 4). At six months postoperatively, MSCT demonstrated further progression with increased metastatic burden, infiltration into the abdominal wall, and invasion of the right psoas muscle. Despite systemic therapy, the patient’s condition progressively deteriorated, and he succumbed to the disease nine months after surgery.

## Discussion

Primary renal neuroendocrine tumors are very rare neoplasms. The spectrum includes well-differentiated NETs (carcinoid tumors), atypical carcinoids, and poorly differentiated neuroendocrine carcinomas such as small cell carcinoma and large cell neuroendocrine carcinoma [2]. Despite their histological diversity, all renal NETs share a propensity for early metastasis, unpredictable behavior, and a challenging clinical course due to the lack of specific symptoms and imaging characteristics [5,10].

Our patient, who was a 24-year-old male individual, presented with hematuria and a massive right renal mass with tumor thrombus extending into the IVC, and was ultimately diagnosed with a high-grade small cell neuroendocrine carcinoma. This presentation is not only rare but also remarkably aggressive for such a young patient. The literature suggests that renal NETs are more commonly diagnosed in the fourth to sixth decades of life, with few reported cases in younger adults [2,8].

IVC involvement is a particularly uncommon and ominous feature in renal NETs. Shantharam et al. reported what may be the first case of a well-differentiated renal NET with IVC tumor thrombus, emphasizing the uniqueness and surgical complexity of such cases [10]. Similarly, Xu et al. described a case of small cell carcinoma of the kidney with a tumor thrombus extending into both the IVC and pulmonary artery, highlighting the aggressive vascular invasion and the need for extensive surgical management [11]. In our case, imaging also revealed multiple intratumoral pseudoaneurysms associated with rapid tumor growth, angiogenesis, and risk of hemorrhage. These pseudoaneurysms may reflect a high degree of vascular remodeling and instability, contributing to the patient's acute presentation with worsening pain and hematuria. During surgery, intraoperative Doppler ultrasound confirmed the extent of the IVC thrombus, which required meticulous thrombectomy and reconstruction, further reflecting the rarity and severity of this condition.

Histologically, small cell carcinoma of the kidney is characterized by sheets of small round cells, high mitotic activity, and necrosis, with strong immunopositivity for neuroendocrine markers such as synaptophysin, CD56, and chromogranin [4,8]. A Ki-67 index of approximately 70%, as seen in our patient, is indicative of a highly proliferative tumor and correlates with poor prognosis [3].

Prognostically, renal NETs display considerable variability depending on differentiation. Well-differentiated tumors may exhibit indolent growth with long-term survival, even in the presence of metastasis [1,13]. In contrast, poorly differentiated tumors, especially small cell variants, are associated with rapid progression and poor outcomes. In the largest population-based study by Omidele et al., patients with small cell carcinoma had the worst median overall survival (7.9 months) compared to other histologies, with surgery being the only significant factor improving survival [3]. Chemoresistance poses a significant challenge in treating SCNC, with regimens such as carboplatin and etoposide typically achieving disease control for only three to six months, highlighting the need for research into targeted therapies and immunotherapy [1,2]. Our patient, despite undergoing timely radical nephrectomy and adjuvant chemotherapy, developed multifocal liver metastases within three months and succumbed to the disease nine months postoperatively, aligning with reported survival expectations.

Another important point is the surprising behavior of some renal NETs: low-grade tumors, like typical carcinoids, can sometimes spread early to lymph nodes or distant organs, while some high-grade tumors may temporarily respond to chemotherapy [10,13]. This unpredictability complicates management strategies and underscores the need for long-term, rigorous surveillance regardless of grade [1,10].

In summary, this case highlights that primary renal NETs, especially small cell types, are exceptionally rare, and aggressive, and may occur in young patients. IVC tumor thrombus is a rare but poor prognostic sign. The presence of intratumoral pseudoaneurysms may reflect an additional marker of vascular aggression and hemorrhagic risk. Given the rarity and complexity of such cases, the prognosis remains poor despite aggressive treatment. Even with surgical resection and systemic chemotherapy, high-grade tumors frequently progress rapidly, with median survival typically less than one year. Comprehensive multidisciplinary management and ongoing molecular research are essential to enhance diagnostic precision and develop more effective therapeutic strategies.

## Conclusions

This case highlights an extremely rare presentation of primary renal small cell neuroendocrine carcinoma with inferior vena cava tumor thrombus in a young patient. Despite radical surgery and systemic chemotherapy, the disease progressed rapidly, leading to death within nine months. It underscores the aggressive nature and poor prognosis of high-grade renal NETs, the significance of IVC involvement, and the urgent need for better diagnostic and therapeutic approaches. Future research should prioritize the development of targeted therapies, immunotherapy strategies, and genomic profiling of renal SCNC to identify actionable mutations and improve clinical outcomes.
